# Antitumor Activity and Mechanism Study of Riluzole and Its Derivatives

**DOI:** 10.22037/ijpr.2020.1101149

**Published:** 2020

**Authors:** Xiang-Long Wu, Liu Liu, Qing-Chuan Wang, Hai-Fang Wang, Xiang-Rong Zhao, Xu-Bin Lin, Wen-Jun Lv, Yin-Bo Niu, Ting-Li Lu, Qi-Bing Mei

**Affiliations:** a *Key Laboratory for Space Bioscience and Biotechnology, School of Life Sciences, Northwestern Polytechnical University, Xi’an, China. *; b *Laboratory Center of Shaanxi Province People's Hospital, Xi'an, China. *; c *Department of Pharmacology, The Fourth Military Medical University, Xi'an, China.*

**Keywords:** Riluzole, Benzothiazole derivatives, Antitumour activity, Synthesis, Mechanism

## Abstract

To explore novel antitumor agents with high efficiency and low toxicity, riluzole alkyl derivatives (**4a-4i**) were synthesized. Their anti-proliferative activities against HeLa, HepG2, SP2/0, and MCF-7 cancer cell lines were assessed by the CCK-8 assay and compared with human normal liver (LO2) cells. Most of them showed potent cytotoxic effects against four human tumor cell lines and low toxic to LO2 cells. In particular, 2-(N-ethylamine)-6-trifluoromethoxy- benzothiazole (**4a**) showed a IC_50_ value of 7.76 μmol/L in HeLa cells and was found to be nontoxic to LO2 cells up to 65 μmol/L. Furthermore, flow cytometry indicated that **4a** could induce remarkable early apoptosis and G2/M cell cycle arrest in HeLa cells. It also impaired the migration ability of HeLa cells in wound healing assays. Western blot results demonstrated that **4a **suppressed Bcl-2 protein expression but increased the level of Bax in HeLa cells, and elevated the Bax/Bcl-2 expression ratio. These new findings suggest that **4a **exhibited beneficially anti-cervical cancer effect on HeLa cells by inducing HeLa cell apoptosis.

## Introduction

Cancer can be defined as a genomic disease that appears as a result of dynamic changes of the DNA of an organism’s cells during its life time. Cancer can develop in almost any organ or tissue. There are many kinds of cancer such as cervical cancer, breast cancer, prostate cancer, skin cancer, lung carcinoma, multiple myeloma, and T cell leukemia (1). Liver cancer is one of the common malignancies in many countries and an increasing cause of cancer death. Breast cancer is also a global concern, accounting for nearly a quarter of female cancer (2). According to the American Cancer Society, approximately one in eight women in the U.S. will develop invasive breast cancer at some point in their life (3). This toll will continue to increase as the number of women in age groups at risk for breast cancer increases. Cervical cancer (CC) is the second leading cause of cancer morbidity and mortality for women around the world. It is the term for a malignant, neoplasm arising from cells originating in the cervix uteri (4, 5).

The major treatments of cancer include surgery, radiation therapy, chemotherapy, immunotherapy, and vaccine therapy. The chemotherapy drug of cancer includes cisplatin, cyclophosphamide, sorafenib, ifosfamide, doxorubicin, and so on (6). However, these molecules display serious side effects in patients treated for a long time. Thus, it is important to develop new medicines and novel approaches to increase the antitumor effects against cancer cells in order to increase the efficacy of cancer treatments. Riluzole (2-amino-6-trifuromethoxybenzothiazole, Compound **1**) has an absolute bioavailability of approximate 60%, which is relatively high, and is also able to cross the blood-brain barrier. Riluzole has been approved and marketed for the treatment of ALS in many countries. In addition, it is also effective in animal models of Parkinson’s disease, Huntington’s disease and cerebral and retinal ischaemia (7). Riluzole exhibits strong anticonvulsant, neuroprotection and antidepressant effects, as well as sedative properties (8).

 Furthermore, increasing evidences have confirmed the anti-nociceptive and anti-allodynic efficacy of riluzole in rat models of spinal cord injury (SCI) and in other pain models (9, 10).

Recently, preclinical studies also have demonstrated anti-tumor effects of riluzole in melanoma (11). Speyer and co-workers reported antitumor properties of riluzole in TNBC, which is an aggressive subtype of breast cancer with a high mortality rate (12). Seol *et al.* described that the riluzole can lead to a suppression of cell proliferation in liver primary cancer cells and cancer cell lines (13). Riluzole can also inhibit proliferation, induce apoptosis and prevent migration of osteosarcoma cells LM7 and glioblastoma cells U87 (14-16). Moreover, riluzole has been observed to reduce the growth of cancer cells in culture or in xenograft models for breast and prostate cancers.

We always focus on the riluzole and its alkyl derivatives for their lipid solubility and extensive physiological activities. Inspired by the antitumor effects of riluzole, we speculated that the alkyl derivatives of riluzole also have antitumor effects. Therefore, in this paper, antitumor properties of riluzole and its alkyl derivatives were evaluated against four cell lines: HeLa (human cervical cancer cell line), HepG2 (human liver cancer cell line), SP2/0 (mouse myeloma cell line), and MCF-7 (human breast cancer cell line). 

## Experimental

*Characterization techniques*

Melting points were measured on a X-4 micro melting point apparatus. IR spectra were recorded on an Equiox-55 FTIR spectrometer. ^1^H &^13^C spectra were recorded on a BRUKER AVANCE instrument. Elemental analyses for C, H, and N were obtained using a Vario EL-III analyzer. All the reagents and solvents used were of analytical grade and were used as supplied unless otherwise stated. TLC was performed on silica gel coated plates for monitoring the reactions.

*Synthesis of compound* ***2*** *.*

6-Trifluoromethoxy-2-amino-benzothiazole (46.82 g, 0.20 mol), hydrazinium sulfate (NH_2_NH_2_• H_2_SO_4_, 39.04 g, 0.30 mol) and hydrazine hydrate (80% aqueous solution, 160 mL) were added to ethylene glycol (500 mL). The mixture was heated and stirred under nitrogen at 130 °C for 4 h. TLC (CH_2_Cl_2_ / MeOH = 20:1) was used to confirm the completion of the reaction. After cooling to room temperature, the mixture was poured into the ice-water. A lot of gray solid was precipitated. The precipitate was filtered and washed three times with water. The solid was dried in vacuum to constant weight.

*Synthesis of compound* ***3*** *.*

Compound **2** (24.90 g, 0.10 mol) was slowly added in portions to a solution of SOCl_2_ (240 mL) which was pre-heated at 65 °C. The solution was stirred for 5 h at 60 °C. Thionyl chloride was evaporated, then the residue was dissolved in CH_2_Cl_2_ and solvent was evaporated again. This process was repeated at least three times in order to remove all SOCl_2_. The residue was dissolved in CH_2_Cl_2_ and washed three times with water, dried over anhydrous Na_2_SO_4_. The solvent was removed under reduced pressure. The crude product was purified by column chromatography on silica gel eluted with petroleum ether (60-90 °C) to give compound **3** (21.42 g, Yield 85%) as a white solid.

*General procedure for the preparation of compounds* ***4a-4i*** *.*

According to our previous research (17), 2-Chloro-6-trifluoromethoxy-benzothiazole (2.54g, 10 mmol) was dissolved in 10 mL ethylamine aqueous solution. The resultant mixture was stirred at room temperature overnight. The solvent was removed under reduced pressure. The residue was dissolved in CH_2_Cl_2_ and washed three times with water, dried over anhydrous Na_2_SO_4_, filtered and the solvent was completely removed. The crude material was purified by column chromatography over silica used dichloromethane as eluent to give compound **4a** as a white amorphous powder (2.22 g, 84.7%). Compounds **4b**-**4i** were also prepared by the same procedure from compound** 3** with *n*-butylamine, diaethylamin, cyclohexylamine, pyrrolidine, piperidine, morpholine, and 4-methylpiperidine, respectively. 

*Cell cultures*

MCF-7 (breast cancer cell line) was cultured in RPMI-1640 medium. HeLa (human cervical cancer cell line), HepG2 (human liver cancer cell line), SP2/0 (mouse myeloma cell line), and LO2 (human normal liver cell line) were cultured in DMEM medium. Each medium was supplemented with 10% fetal bovine serum, 1% L-glutamine and 1% penicillin/streptomycin solution. The cell lines were kept in the incubator at 37 ℃ in a humidified atmosphere (90% RH) containing 5% CO_2_. The optimal plating density of cell lines was determined to be 5×10^4^. The cell lines SP2/0 and MCF-7 were obtained from Laboratory Center of Shaanxi Province People’s Hospital.

*In-vitro anti-proliferative activity assays*

All of the synthesized compounds (**1-3** and **4a-4i**) were assessed for their cytotoxic activity by Cell Counting Kit-8 (Beyotime, China) using the HeLa, HepG2, SP2/0, MCF-7 and LO2 cell lines. 2×10^3^ cells per well were planted into 96-well plates in 100 μL growth medium and incubated for 24 h. Then, the cells were exposed to each compound in growth medium (DMSO< 0.5%) at five different concentrations (1.23, 3.70, 11.11, 33.33, and 100 μmol/L) for 48 h. The mixture was incubated at 37 ℃ under a humidified 5% CO_2_ atmosphere. After incubation, 10 μL CCK-8 solution was added to each well and the cells were further incubated for 2 h at 37 ℃ according to the CCK-8 Technical Manual. Then, the OD value for each well was read at wavelength 450 nm to determine the cell viability on a microplate reader (Synergy HT, USA). The IC_50_ (50% cell viability inhibition) values were calculated by means of SPSS, GraphPad prism software. The independent experiment was repeated at least three times. The percentage of growth inhibition was calculated as follows according to reference (18),

% of growth inhibition = [1 - absorbance of treated cells/absorbance of untreated cells] × 100%

*Measurement of anti-migration activity*

Effects of compound **2 **and **4a **on the cell migration were evaluated in monolayer of HeLa cells. Once the monolayer was formed, a small scratch was made in the plate using a 10 μL pipette tip (0 h, day 1). The monolayer was once washed with PBS to remove debris or the detached cells from the monolayer. The compound **2** and **4a** were treated at 25 μmol/L concentration and observed the plates after 24 h (day 2). Images were taken using an inverted microscope (19). The experiments were performed in triplicate.

The relative scratch healing rate was calculated as follows according to reference (20),

Scratch healing rate = (scratch width at 0 h – the remaining scratch width at 24 h)/scratch width in 0 h×100%

*Cell morphological assessment*

The cell morphological assessment was carried out as described by Wang S (21). Morphological changes, such as chromosomal condensation and fragmentation in the nuclei of HeLa, were observed by Hoechst 33258 staining. In brief, HeLa cells were seeded at a concentration of 1×10^6^ cells/well in 6-well plate and treated with compound **4a **(12.5, 25 and 50 μmol/L) for 24 h. Subsequently, the cells were harvested and washed once with PBS and then fixed with 4% paraformaldehyde in PBS for 10 min. After staining with 10 mg/L Hoechst 33258 for 5 min, the cells were visualized under a fluorescent microscope (Nikon, Japan). Viable cells displayed normal nuclear size and uniform fluorescence, whereas apoptotic cells showed condensed, fractured, or distorted nuclei (22).

*Flow cytometric analysis of the cell cycle*

Cell cycle assay was performed using the cell cycle and apoptosis analysis kit (Beyotime, China) and following the manufacturer’s instructions. The HeLa cells were treated with the indicated concentrations of compound **4a** (12.5, 25 and 50 μmol/L) for 24 h. Next, the cells were harvested and washed twice with PBS. The cells were fixed with cold 70% ethanol for 12 h at 4 ℃ and were harvested and centrifuged at 1000 g for 5 min. Then, the cell density was adjusted to 1×10^6 ^cells/mL with PBS. Finally, the cells were stained with a propidium iodide (PI) solution (50 μg/mL). The number of cells in each phase of the cell cycle was analyzed by flow cytometry (FACSCalibur; BD Bioscience, USA).

*Flow cytometric analysis of cell apoptosis*

The cell apoptosis analysis was evaluated by using the annexin V-FITC apoptosis detection kit (Beyotime, China). Briefly, the HeLa cells (1×10^6 ^cells/well) were seeded into 6-well culture plates and incubated for 24 h. Then the cells were incubated for 24 h with the compound **4a **(12.5, 25 and 50 μmol/L). The cells were then rinsed, resuspended in 60 μL binding buffer and incubated for 10 min with 5 μL annexin V-FITC at room temperature, followed by an addition of 120 μL binding buffer and 10 μL PI. The solutions were gently mixed and analyzed with the FACScan flow cytometry. The cells that were not treated were used as the control (23).

*Western blot assay*

HeLa cells were seeded into 6-well plates at 10^6^ cells per well with different concentrations of compound **4a **(12.5, 25 and 50 μmol/L) and incubated at 37 ℃ for 24 h in the presence of 10% FBS. The controls were treated with vehicle (0.5% DMSO). The cells were lysed into ice-cold cell lysis buffer for 30 min and centrifuged at 13000 g for 15 min at 4 ℃. The protein concentrations of the supernatant were determined by a BCA protein Assay Kit. Equal amounts of protein lysates were separated on 10% SDS-PAGE (sodium dodecyl sulfate-polyacrylamide gel electrophoresis) and transferred onto PVDF membranes. The membranes were blocked with 5% non-fat milk in TBST (0.1% Tween 20 in TBS) buffer for 2 h, incubated with primary antibodies at 1:1000 dilutions at 4 ℃, and washed by TBST four times. Then, the membrane was immunobloted with Bax, Bcl-2, Caspase-3, Cleaved caspase-3, PI3K, and β-actin primary antibodies at 1:1000 dilutions at 4 °C for 12 h. After secondary incubation in horseradish peroxidase-conjugated secondary antibodies at 1:3000 at room temperature for 2 h, protein bands were visualized on X-ray film (Kodak, Japan) using ECL (Beyotime, China). The immunoblots were detected by densitometric analysis. Equal loading of protein in each lane was confirmed by probing with β-actin antibody (24). 

## Result and Discussion

**Scheme 1:**
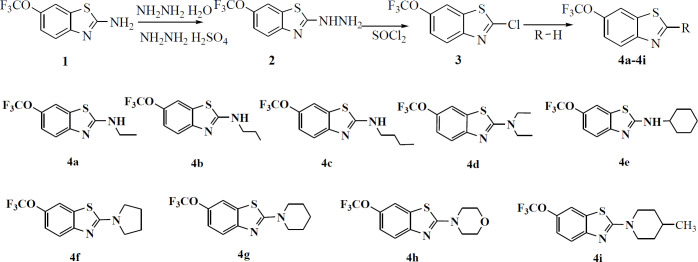
The synthetic procedure of riluzole derivatives and their structures

In order to obtain new compounds, nine N-alkylated derivatives of riluzole were synthesized according to the synthetic procedure shown in Scheme 1. Riluzole was firstly transformed into (6-trifluoromethoxy-benzothiazol-2-yl)-hydrazine, then it was chlorinated by SOCl_2_ to obtain 2-chloro-6-trifluoromethoxy-benzothiazole. This intermediate product was treated with nine alkylamines to give N-alkylated derivatives of riluzole respectively. The structures of compounds were confirmed by means of elemental analysis, IR, ^1^HNMR and ^13^CNMR. The purity of each compound was determined by HPLC analysis. The values of purity were from 98.06% to 100%, which could meet the requirements of biological experiments. The data of these compounds were given as follows.

*Trifluoromethoxy-benzothiazol-2-yl)-hydrazine (Compound 2)*

Gray schistose; mp 205-206 °C; IR (KBr) υ_max_ 3362, 3205, 3124, 2963, 2880, 2361, 1660, 1564, 1464, 1261, 1123 cm^-1^; ^1^H NMR (DMSO-d_6_, 500 MHz): δ= 9.22 (1H, s, -NH-), 7.79 (1H, s, Ar-H), 7.35 (1H, d, *J *= 8.7 Hz, Ar-H), 7.17 (1H, d, *J *= 8.7 Hz, Ar-H), 5.12 (2H, s, -NH_2_); ESI-MS m/z 248.39[M-H]^-^; C_8_H_6_F_3_N_3_OS (calcd. 249.21) Anal. Calcd. for C_8_H_6_F_3_N_3_OS: C, 38.56; H, 2.43; N, 16.86. Found: C, 38.44; H, 2.34; N, 16.75. Its purity by HPLC was 100%.

*Chloro-6-trifluoromethoxy benzothiazole* *(Compound 3)*

White solid; mp 38-39 °C; IR (KBr) υ_max_ 3099, 3078, 2361, 1480, 1452, 1262, 1164, 1016, 865 cm^-1^; ^1^H NMR (CDCl_3_, 500 MHz) : δ= 7.96 (1H, d, *J *= 8.9 Hz, Ar-H), 7.66 (1H, s, Ar-H), 7.37 (1H, d, *J *= 8.9 Hz, Ar-H); ESI-MS: m/z 251.91 [M-H]^-^; C_8_H_3_ClF_3_NOS (calcd.252.96); Anal. Calcd for C_8_H_3_ClF_3_NOS: C, 37.88; H, 1.19; N, 5.52. Found: C, 37.95; H, 1.13; N, 5.59. Its purity by HPLC was 98.06%.

*(N-Ethylamine)-6-trifluoromethoxy-benzothiazole (Compound 4a)*

White solid; mp 133-134 °C; IR (KBr) υ_max_ 3210, 2987, 2922, 2361, 1622, 1584, 1461, 1250, 1214, 1152, 807 cm^−1^; ^1^H NMR (CDCl_3_, 500 MHz): δ = 7.48 (1H, d, *J *= 8.8 Hz, Ar-H), 7.46 (1H, s, Ar-H), 7.16 (1H, d,* J *= 8.8 Hz, Ar-H), 5.69 (1H, s, -NH-), 3.47 (2H, q, *J *= 7.2 Hz, -CH_2_-), 1.34 (3H, t, *J *= 7.2 Hz, -CH_3_); ^13^C NMR(CDCl_3_, 125 MHz): δ = 167.95 (C, S-C-N), 150.94 (C, Ar-O), 143.57 (C, Ar-N), 130.86 (C, Ar-S), 121.65(C, -CF_3_), 119.68 (C, Ar-4), 118.92 (C, Ar-5), 114.07 (C, Ar-7), 40.43 (C, N-C-C), 14.83(C, -CH_3_); Anal. Calcd for C_10_H_9_F_3_N_2_OS: C, 45.80; H, 3.46; N, 10.68. Found: C, 45.93; H, 3.40; N, 10.76. Its purity by HPLC was 100%.

*(N-Propylamine)-6-trifluoromethoxy-benzothiazole (4b)*

White solid; mp 88-89 °C; IR (KBr) υ_max_ 3150, 2978, 2861, 2361, 1614, 1556, 1463, 1281, 1214, 1162, 808 cm^−1^; ^1^H NMR (CDCl_3_, 500 MHz): δ = 7.47 (1H, d, *J *= 8.8 Hz, Ar-H), 7.45 (1H, s, Ar-H), 7.16 (1H, d, *J *= 8.8 Hz, Ar-H), 5.75 (1H, s, -NH-), 3.39 (2H, t, *J *= 7.1 Hz, NH-CH_2_), 1.75-1.69 (2H, m, -CH_2_CH_2_CH_3_), 1.02 (3H, t, *J *= 7.4 Hz, -CH_3_); ^13^C NMR (CDCl_3_, 125 MHz): δ = 168.23 (C, S-C-N), 150.98 (C, Ar-O), 143.54 (C, Ar-N), 130.84 (C, Ar-S), 121.65(C, -CF_3_), 119.68 (C, Ar-4), 118.87 (C, Ar-5), 114.06 (C, Ar-7), 47.44 (C, NH-C-C), 22.79 (C, C-C-C),11.34 (C, -CH_3_); Anal. Calcd for C_11_H_11_F_3_N_2_OS: C, 47.82; H, 4.01; N, 10.14. Found: C, 47.69; H, 4.20; N, 10.03. Its purity by HPLC was 100%.

*(N-n-butylamine)-6-trifluoromethoxy-benzothiazole (4c)*

White solid; mp 76-78 °C; IR (KBr) υ_max_ 3211, 2923, 2859, 2361, 1626, 1566, 1462, 1264, 1166, 815 cm^−1^; ^1^H NMR (CDCl_3_, 500 MHz): δ = 7.47 (1H, d,* J *= 8.8 Hz, Ar-H), 7.45 (1H, s, Ar-H), 7.15 (1H, d, *J *= 8.7Hz, Ar-H), 5.74 (1H, s, -NH-), 3.41 (2H, t, *J *= 7.1 Hz, NHCH_2_), 1.71-1.65(2H, m, NHCH_2_CH_2_), 1.48-1.41 (2H, m, -CH_2_CH_3_), 0.96 (3H, t, *J *= 7.4 Hz, CH_3_); Anal. Calcd for C_12_H_13_F_3_N_2_OS: C, 49.65; H, 4.51; N, 9.65. Found: C, 49.54; H, 4.46; N, 9.69. Its purity by HPLC was 100%.

*(N-diaethylamine)-6-trifluoromethoxy-benzothiazole (4d)*

White solid; mp 38-39 °C; IR (KBr) υ_max_ 3090, 2981, 2938, 2857, 2361, 1607, 1550, 1460, 1359, 1294, 1215, 1151 cm^−1^; ^1^H NMR (Acetone-d_6_, 500 MHz): δ = 7.72 (1H, s, Ar-H), 7.46 (1H, d, *J *= 8.8 Hz, Ar-H), 7.21 (1H, d, *J *= 8.8Hz, Ar-H), 3.62 (4H, q, *J *= 7.1 Hz, 2×-CH_2_-), 1.27 (6H, t, *J *= 7.1 Hz, 2×-CH_3_); ^13^C NMR (CDCl_3_, 100 MHz): δ = 167.81 (C, S-C-N), 152.15 (C, Ar-O), 142.94 (C, Ar-N), 131.27 (C, Ar-S), 121.94(C, -CF_3_), 119.50 (C, Ar-4), 118.64 (C, Ar-5), 113.76 (C, Ar-7), 45.52 (C, N-C-C), 12.80 (C, -CH_3_); Anal. Calcd for C_12_H_13_F_3_N_2_OS: C, 49.65; H, 4.51; N, 9.65. Found: C, 49.54; H, 4.62; N, 9.77. Its purity by HPLC was 98.45%.

*(N-cyclohexylamine)-6-trifluoromethoxy-benzothiazole (4e)*

White solid; mp 96-97 °C; IR (KBr) υ_max_ 3427, 3231, 2933, 2858, 1616, 1548, 1456, 1249, 1220, 1162 cm^−1^; ^1^H NMR (CDCl_3_, 500 MHz): δ = 7.46 (1H, d, *J*=8.8 Hz, Ar-H), 7.44 (1H, s, Ar-H), 7.15 (1H, d, *J* = 8.7Hz, Ar-H), 5.53 (s, 1H, -NH-), 3.56 (1H, t, *J *= 9.6 Hz, -CH-), 2.14-2.10(2H, m, -CH_2_-), 1.80-1.76 (2H, m, -CH_2_-), 1.52-1.14 (6H, m, 3×-CH_2_-); ^13^C NMR(CDCl_3_, 100 MHz): δ = 167.12 (C, S-C-N), 151.26 (C, Ar-O), 143.45 (C, Ar-N), 130.98 (C, Ar-S), 121.94(C, -CF_3_), 119.62 (C, Ar-4), 118.85 (C, Ar-5), 113.98 (C, Ar-7), 54.64 (C, N-C-C), 33.20 (C, -CH_2_-), 25.42 (C, -CH_2_-), 24.68 (C, -CH_2_-); Anal. Calcd for C_14_H_15_F_3_N_2_OS: C, 53.15; H, 4.78; N, 8.86. Found: C, 53.33; H, 4.68; N, 8.67. Its purity by HPLC was 99.28%.

*(N-pyrrolidine)-6-trifluoromethoxy-benzothiazole (4f)*

White solid; mp 129-130 °C; IR (KBr) υ_max_ 3418, 2969, 2870, 2361, 1614, 1554, 1452, 1365, 1243, 1214, 1184 cm^−1^; ^1^H NMR (CDCl_3_, 500 MHz): δ = 7.53 (1H, d, *J *= 8.8 Hz, Ar-H), 7.46 (1H, s, Ar-H), 7.15 (1H, d, *J *= 8.8 Hz, Ar-H), 3.58 (4H, t, *J*=6.3Hz, CH_2_-N-CH_2_), 2.11-2.06 (4H, m, -CH_2_CH_2_-); ^13^C NMR (CDCl_3_, 100 MHz): δ = 165.79 (C, S-C-N), 152.15 (C, Ar-O), 142.91 C, Ar-N), 131.35 (C, Ar-S), 121.94(C, -CF_3_), 119.49 (C, Ar-4), 118.74 (C, Ar-5), 113.89 (C, Ar-7), 49.52 (C, N-C-C), 25.61 (C, -CH_2_-); Anal. Calcd for C_12_H_11_F_3_N_2_OS: C, 49.99; H, 3.85; N, 9.72. Found: C, 49.87; H, 3.75; N, 9.61. Its purity by HPLC was 100%.

*2-(N-piperidine)-6-trifluoromethoxy-* *benzothiazole (4g)*

White solid; mp 74-75 °C; IR (KBr) υ_max_ 3423, 2940, 2859, 2361, 1608, 1549, 1461, 1246, 1158 cm^−1^; ^1^H NMR (CDCl_3_, 500 MHz): δ = 7.49 (1H, d, *J*=8.8 Hz, Ar-H), 7.45 (1H, s, Ar-H), 7.14 (1H, d, *J *= 8.8 Hz, Ar-H), 3.61-3.60 (4H, m, -CH_2_-N-CH_2_-), 1.72-1.71 (6H, m, -CH_2_CH_2_CH_2_-); ^13^C NMR (CDCl_3_, 125 MHz): δ = 169.27 (C, S-C-N), 151.78 (C, Ar-O), 143.17 (C, Ar-N), 131.32 (C, Ar-S), 121.69(C, -CF_3_), 119.56 (C, Ar-4), 118.92 (C, Ar-5), 113.80 (C, Ar-7), 49.68 (C, N-C-C), 25.27 (C, -CH_2_-), 24.17 (C, -CH_2_-); Anal. Calcd for C_13_H_13_F_3_N_2_OS: C, 51.65; H, 4.33; N, 9.27. Found: C, 51.46; H, 4.42; N, 9.16. Its purity by HPLC was 100%.

*(N-morpholine)-6-trifluoromethoxy-benzothiazole (4h)*

White solid; mp 103-105 °C; IR (KBr) υ_max_ 3447, 2974, 2907, 2866, 1608, 1549, 1459, 1380, 1339, 1267, 1112, 1026 cm^−1^; ^1^H NMR (CDCl_3_, 500 MHz): δ = 7.52 (1H, d, *J*=8.8 Hz, Ar-H), 7.49 (1H, s, Ar-H), 7.17 (1H, d, *J *= 8.8 Hz, Ar-H), 3.84-3.82 (4H, m, -CH_2_-O-CH_2_-), 3.63-3.61 (4H, m, -CH_2_-N-CH_2_-); ^13^C NMR (CDCl_3_, 125 MHz): δ = 169.45 (C, S-C-N), 151.22 (C, Ar-O), 143.64 (C, Ar-N), 131.19 (C, Ar-S), 121.65(C, -CF_3_), 119.86 (C, Ar-4), 119.57 (C, Ar-5), 113.98 (C, Ar-7), 66.18 (C, N-C-C), 48.51 (C, -CH_2_-); Anal. Calcd for C_12_H_11_F_3_N_2_O_2_S: C, 47.37; H, 3.64; N, 9.21. Found: C, 47.49; H, 3.54; N, 9.30. Its purity by HPLC was 100%.

*2-(N-4-methyl-1-piperidinyl)-6-trifluoromethoxy-benzothiazole (4i)*

White solid; mp 77-78 °C; IR (KBr) υ_max_ 3419, 2940, 2805, 2727, 1614, 1470, 1358, 1271, 1184, 772 cm^−1^;^ 1^H NMR (CDCl_3_, 500 MHz): δ = 7.47 (1H, d, *J* = 8.7 Hz, Ar-H), 7.44 (1H, s, Ar-H), 7.13 (1H, d, *J* = 8.2 Hz, Ar-H), 4.09 (2H, d, *J* = 12.7 Hz, N-CH_2_-), 3.12 (2H, t, *J* = 12.7 Hz, -CH_2_-N), 1.77 (2H, d, *J* = 12.7 Hz, -CH_2_-), 1.68–1.63 (1H, m, -CH-), 1.36-1.26 (2H, m, -CH_2_-), 1.00 (3H, d, *J* = 6.5 Hz, -CH_3_); ^13^C NMR (CDCl_3_, 100 MHz): δ = 169.20 (C, S-C-N), 151.80 (C, Ar-O), 143.18 (C, Ar-N), 131.36 (C, Ar-S), 121.93 (C, -CF_3_), 119.60 (C, Ar-4), 118.94 (C, Ar-5), 113.83 (C, Ar-7), 49.07 (C, N-C-C), 33.43 (C, -CH_2_-), 30.79 (C, -CH-), 21.71 (C, -CH_3_); Anal. Calcd for C_14_H_15_F_3_N_2_OS: C, 53.15; H, 4.78; N, 8.86. Found: C, 53.76; H, 4.60; N, 8.70. Its purity by HPLC was 100%.

We optimized the synthetic condition and processing method of compound **3** based on a reference (25). For example, in the preparation process of compound **2**, the precipitate was vacuum filtered and triturated in a mixture of water-diethyl ether according to the reference (26). Because precipitate was not complete, the yield was very low. Therefore, our method was that the reaction mixture was poured into ice water. In the process of preparing compound **3**, it was not an appropriate method to destroy the residue SOCl_2 _by water. The SOCl_2 _should be evaporated, then the residue was dissolved in CH_2_Cl_2_. The CH_2_Cl_2_ which contains residual SOCl_2_ was evaporated again. It can be used repeatedly. In the second step reaction, tail gas absorption equipment was needed. As we can see from scheme 1, compound **3 **was an important intermediate. The chlorine atom in compound **3** had high reactivity, so it was very easy to be substituted by the alkylamine. In the third step reaction, the alkylamine was not only a substrate but also a solvent. The excess alkylamine could be recycled under reduced pressure after the completion of the reaction. Therefore, the solvent was economized owing to its reused characteristics and the reduction of environmental pollution.

These compounds were screened for their anti-proliferative activities in four tumor cell lines (HeLa, HepG2, SP2/0 and MCF-7) using CCK-8 assay. The resulting cytotoxic activity data of riluzole and its derivatives were presented in Table 1.

Regarding the series of riluzole derivatives, the highest activity was displayed by compound **4a** in HeLa and MCF-7 cancer cells (IC_50 _= 7.76 and 7.72 μmol/L, respectively). It is very close to the reference drug Sorafenib. Compound **4b **obviously reduced HepG2 cell proliferation and IC_50_ value was found to be 17.97 μmol/L. Compound **4b** was the best one among all compounds for HepG2 cell. In the SP2/0 cancer cells, the highest activity was observed for compound **2 **(IC_50_ = 7.45 μmol/L). Interestingly, compound **2 **with hydrazine substituent had good activity (IC_50_ in the range of 7.45–28.58 μmol/L) in all the examined cancer cell lines whereas compound **4i** with N-4-methyl-1-piperidinyl substituent was found potent (IC_50 _= 11.52 μmol/L) only in MCF-7 cancer cell line. Riluzole had some activities in all the examined cancer cell lines; however, the IC_50_ values (the range of 29.14 – 48.38 μmol/L) were higher than some derivatives.

Anti-proliferative activities of compounds **3**, **4f**, and **4h **were negligible because their IC_50_ values were more than 100 μmol/L, which were above the highest clinically attainable concentration. The structure activity relationship (SAR) couldn’t be concluded, because different activities for these compounds were shown for different cancer cells. For these four cancer cell lines, the highest activity compound for each one was found, such as **4a ***vs *HeLa and MCF-7, **4b ***vs *HepG2, compound **2 ***vs *SP2/0. 

**Figure 1:**
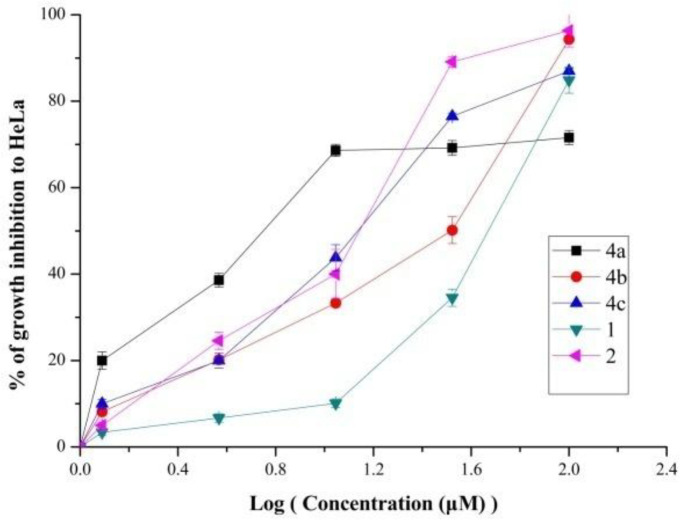
The relationship between growth inhibition and concentration of compounds **1**, **2**, **4a**-**4c** on HeLa cells

The relationship between growth inhibition and concentration of some compounds (**1**, **2**, **4a**-**4c**) were investigated. Results depicted in Figure 1. indicated that these five compounds reduced HeLa cell proliferation in a dose-depended manner. As we can see from the Figure 1, the percentage of growth inhibition of **4a **increased rapidly as the concentration increase. However, other compounds especially riluzole increased slowly. These results also suggested that compound **4a** had conspicuously anti-proliferative activity.

The cytotoxic effects were also researched in the normal human liver (LO2) cells to assess the toxicity of these compounds (Table 2). The selectivity index (SI) was calculated as the ratio of the IC_50_ for the normal cell line (LO2) to the IC_50_ for a respective cancerous cell line. Higher values of SI indicate greater anticancer specificity and the compounds displaying SI values higher than 3 were considered to be highly selective (28). Some of riluzole derivatives not only had high cytotoxic activity against cancer cells but also displayed low toxicity against normal human liver (LO2) cells and their SI values were higher than 3.5. The SI values of compound **4a** in HeLa and MCF-7 cancer cells were 8.37 and 8.41, respectively. The SI value of compound **2** in SP2/0 cancer cells was as high as 13.42, which had shown greater anticancer specificity. In this paper, compound **4a** was investigated in further research for its excellent anticancer activity.

**Figure 2:**
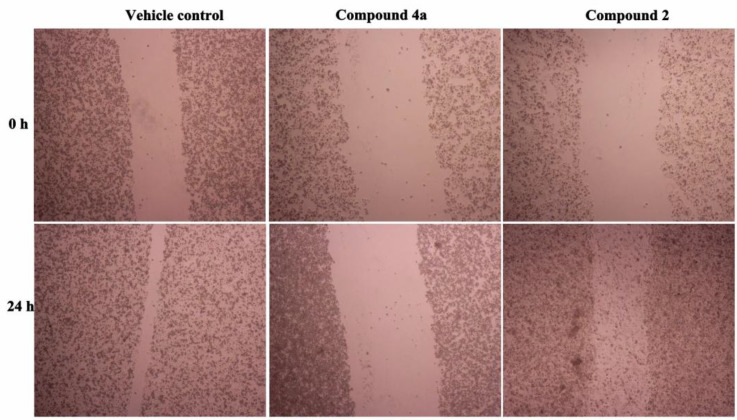
Effects of compounds** 2 **and** 4a **on cell migration by wound healing assay (25μmol/L). Upper panel: **Vehicle**, **4a** and **2** groups at 0-h time point. Bottom panel: **Vehicle**, **4a** and **2** groups at 24-h time point

Cancer cell migration and invasion is the main feature responsible for malignant tumor progression and metastasis (29). Cellular migration, the major process in cancer metastasis starts with the loss of cell-cell adhesion (helps in the detachment of cells from primary tumor) followed by loss of cell-matrix interaction (drives the cells to invade to the surrounding stroma) (30). To investigate the effect of compound **2** and **4a **on the cell migration ability, we performed the vitro scratch wound healing assay by using HeLa cancer cells. As shown in Figure 2, the wound gap of control group (vehicle) almost closed after 24 h without treatment. However, the gap width of **4a** group didn’t change after treatment with compound **4a** for 24 h. HeLa cell migration was significantly slower in the **4a** group than in the control group. 

**Figure 3:**
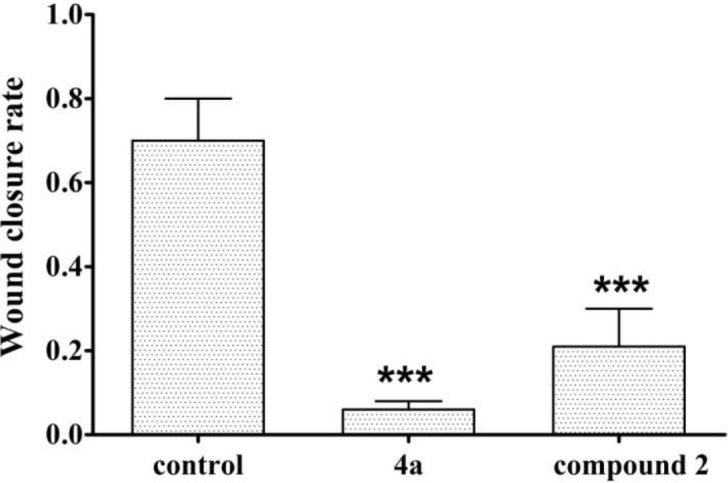
Anti-migration scratch healing rate of compounds **2**, **4a** on HeLa cells (N = 3, ***p < 0.01).

As shown in Figure 3, the relative scratch healing rate of compound **4a** was less than 20%, the scratch healing rate of compound **2 **was less than 40%, and the wound healing rate of blank group was as high as 70%. So compound **4a** had significant anti-migration effect which indicates that it has the potential to inhibit HeLa cell metastasis *in-vivo*. The data from the scratch assay demonstrated that compound **2** could also prevented migration of HeLa cells.

The concentration was an important factor. If the concentration was too high, the toxicity was so strong that the monolayer of cell will disappear. However, the effect on the migration can’t be observed in low concentration. Three drug concentrations (12.5, 25 and 50 μmol/L) were chosen in our experiment and the 25 μmol/L was the best one.

**Figure 4:**
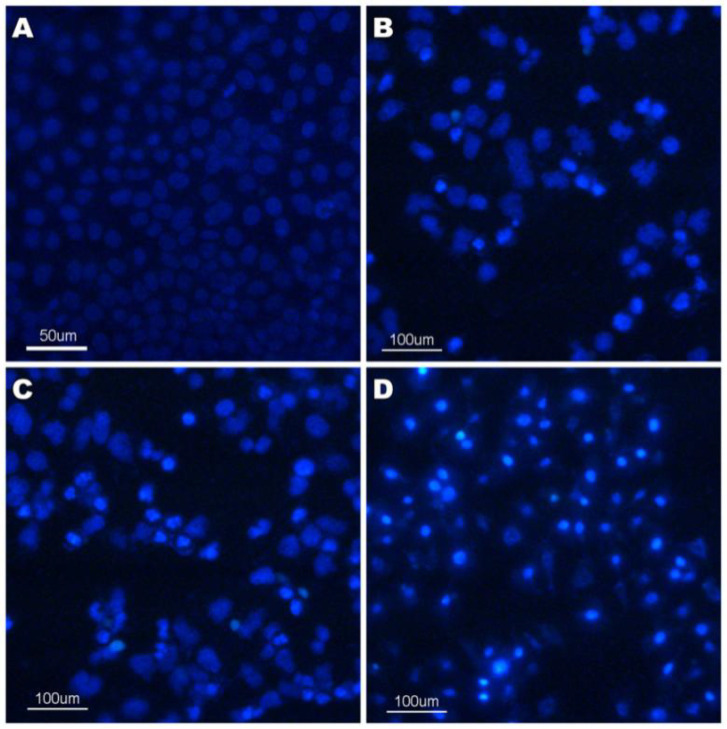
Hoechest 33258 staining of compound **4a** in HeLa cell line. (A)Control; (B) 12.5 μmol/L** 4a** for 24 h; (C) 25 μmol/L** 4a** for 24 h; (D) 50 μmol/L** 4a** for 24 h

The changes of morphological features, such as cell shrinkage, chromatin condensation, and nuclear membrane blebbing were the characteristics of apoptotic cells (31). The morphological assay of cell death was investigated by Hoechst 33258 staining. Hoechst 33258, which stains the cell nuclei and emits fluorescence allowing the visualization of nuclear morphological changes, was a membrane permeable dye. We had observed the morphological changes associated with the cells upon the treatment with compound **4a** using fluorescence microscopy. The results were given in Figure 4. The results showed that the control cells were normal and the nuclei were round and homogeneous. However, the nuclei treated with compound **4a** for 24 h exhibited nuclear condensation and fragmentation which is the typical characteristics of apoptosis. This phenomenon was observed in a dose-dependent manner as we can see from the Figure 4.

**Figure 5:**
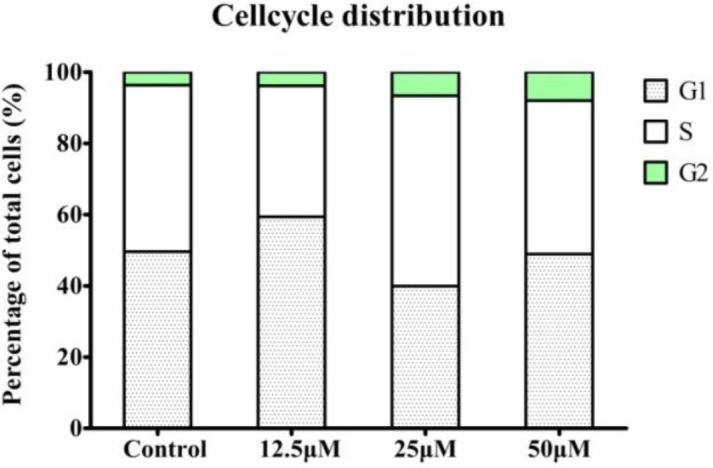
Effect of compound **4a** on cell cycle. The distribution of HeLa cells in the three phases of the cell cycle following treatment with compound **4a **is depicted in representative plots

To further investigate the mechanism of compound **4a **on HeLa, we examined the effect of **4a** on cell cycle distribution by flow cytometry. As shown in Figure 5, the cells in the G2/M phase increased from 3.66% in control group to 3.85%, 6.63%, and 8.00% in a concentration-dependent manner in HeLa cell lines. These results revealed that compound **4a** dose-dependently arrested the cell cycle at the G2/M phase, thereby reducing the proportion of cells in the S and G1 phase.

**Figure 6:**
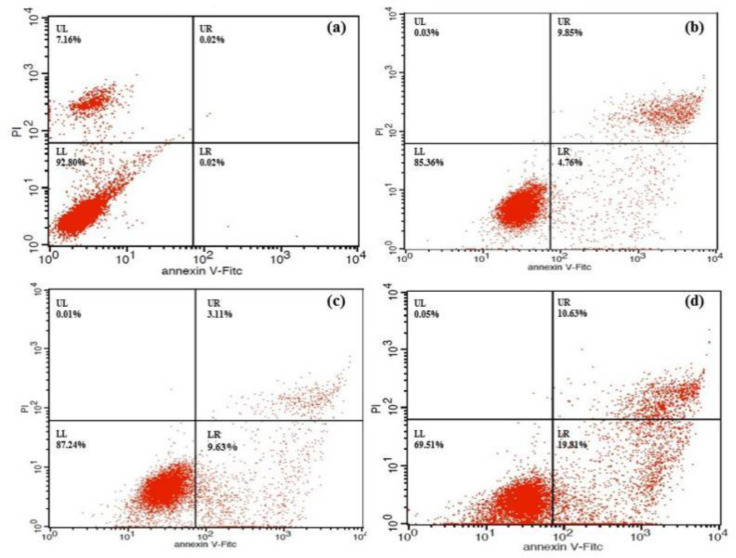
Flow cytometric analysis of HeLa cells treated with compound 4a. The percentages of apoptotic cells are shown in representative plots. (a) Control (b)12.5 μmol/L (c) 25 μmol/L (d) 50 μmol/L

**Figure 7:**
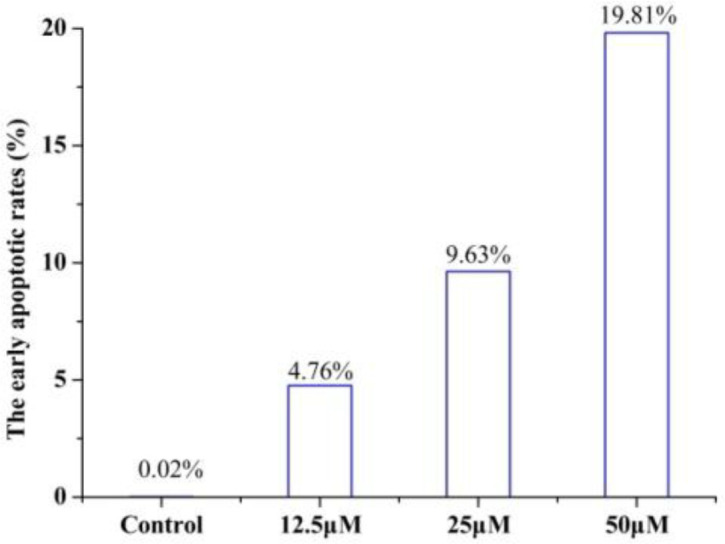
Effect of compound **4a** on the early apoptotic rates (μM = μmol/L).

To determine whether the growth inhibitory effect of **4a** was associated with cell apoptosis, we performed annexin V-FITC/PI double-staining and flow cytometry of HeLa cells. The early apoptotic rates were 4.76 %, 9.63 %, and 19.81% at **4a** concentrations of 12.5, 25 and 50 μmol/L, respectively (Figure 6). Compound **4a** could induce remarkable early apoptosis of HeLa in a dose-dependent manner (Figure 7). These results suggested that the inhibition of cell growth was caused by the induction of early apoptosis.

**Figure 8:**
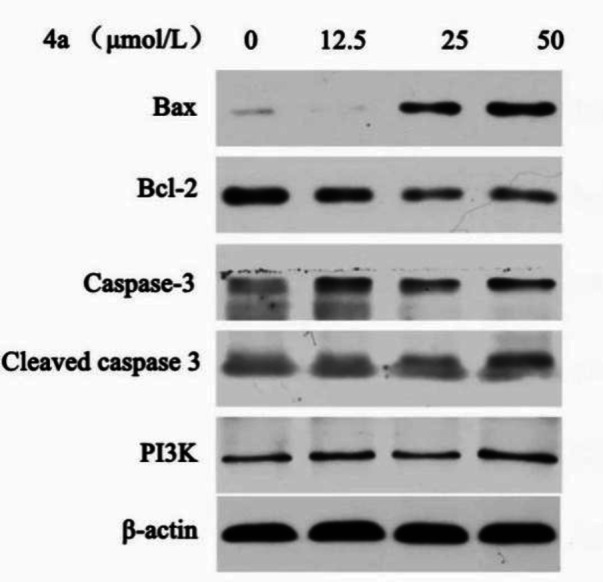
The effects of compound **4a** on the protein levels were assessed by Western blot

**Figure 9:**
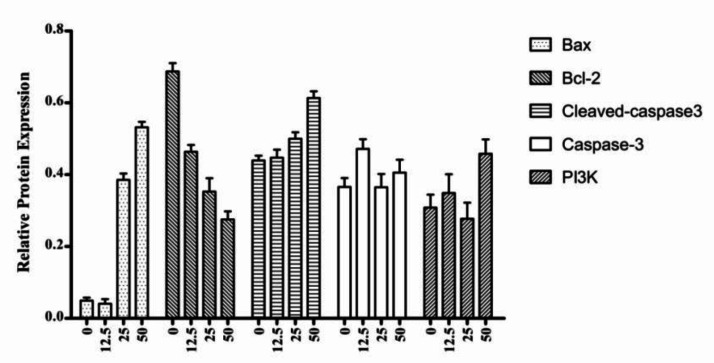
The fold-changes in the relative protein levels were calculated with reference to the controls

The Bcl-2 family proteins can either positively or negatively regulate apoptosis. The pro-apoptotic family members include Bax, Bad and Bok, while the anti-apoptotic members of this family include Bcl-2, Bcl-XL, and Bcl-w (32, 33). We examined the effects of **4a** on the expression of Bax, Bcl-2, Caspase-3, Cleaved caspase-3, and PI3K by western blot analysis (Figure 8). As shown in Figure 9, western blot results demonstrated that **4a** reduced the expression of Bcl-2, but increased the levels of Bax and Cleaved caspase-3 in HeLa cells. There were not remarkably concentration-dependent change in the expression levels of PI3K and Caspase-3 in HeLa cells. As we can see from Figure 8 and Figure 9, Cleaved caspase-3 which is an apoptotic marker was clearly increased in the HeLa cells.

The release of cytochrome c from mitochondria to cytosol is one of the early events prior to apoptosis. And it is widely accepted that the release of cytochrome c into cytosol is tightly regulated by the ratio between the Bcl-2 family proteins, especially the anti-apoptotic protein (Bcl-2) and pro-apoptotic protein (Bax) protein, all of which have been demonstrated to be responsible for the regulation of the apoptotic process (34). As we can see from Figure 9, compound **4a** could inhibit Bcl-2 and induce Bax expression. Furthermore, the increase of the Bax/Bcl-2 

**Table 1:** *In-vitro *cytotoxic activity of all compounds in HeLa, HepG2, SP2/0, MCF-7 and LO2 cell lines

**Compounds**	**Cytotoxicity (IC**_50_ **, μM）**				
	**HeLa**	**HepG2**	**SP2/0**	**MCF-7**	**L02**
**1**	48.38±1.89	43.49±1.72	30.94±1.83	29.14±1.70	84.6±4.70
**2**	10.36±0.77	28.54±0.91	7.45±0.71	10.52±0.66	100±5.16
**3**	>100	>100	>100	>100	100±6.07
**4a**	7.76±0.45	33.69±1.04	51.15±2.73	7.72±0.53	65±4.38
**4b**	30.13±1.28	17.97±0.88	46.44±3.62	12.9±0.69	32.2±3.03
**4c**	16.71±0.94	>100	58.96±3.32	21.94±0.97	25.6±3.12
**4d**	>100	>100	48.46±3.41	46.6±2.77	227±7.82
**4e**	20.94±0.98	20.21±0.93	17.73±0.81	20.49±1.09	33.8±2.40
**4f**	>100	>100	>100	>100	65.2±3.53
**4g**	>100	>100	32.58±1.44	25.8±1.21	134±5.18
**4h**	>100	>100	98.74±3.28	>100	137±5.28
**4i**	>100	>100	11.52±0.85	>100	112±5.62
**Sorafenib**	5.16±0.62	12.04±2.12	4.25±0.75	7.66±0.73	11.91±0.32

**Note**: IC_50_ is the compound concentration required to inhibit cell growth by 50%. Sorafenib was used as a positive control and reference drug (27).

**Table 2:** The calculated values of the selectivity index (SI) of some compounds

**Compounds**	**SI**			
	**HeLa**	**HepG2**	**SP2/0**	**MCF-7**
**1**	1.75	1.95	2.73	2.90
**2**	9.65	3.50	13.42	9.51
**4a**	8.37	1.93	1.27	8.41
**4b**	1.07	1.79	0.69	2.50
**4c**	1.53	0.26	0.43	1.17
**4e**	1.62	1.67	1.91	1.65
**4g**	1.34	1.34	4.12	5.20
**4i**	1.12	1.12	9.68	1.12

**Note: **The selectivity index (SI) was calculated for each compound using the formula: SI = IC_50 _for normal cell line LO2/IC_50_ for respective cancerous cell line. A beneficial SI > 1.0 indicates a compound with efficacy against tumor cells greater than the toxicity against normal cells.

**Figure 10:**
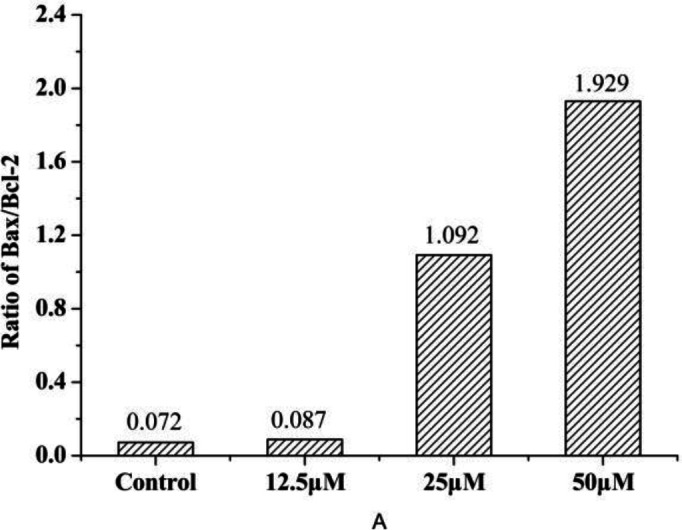
The ratio of Bax and Bcl-2 of HeLa cells treated with compound **4a**. (μM = μmol/L).

## References

[1] Ozcan AG (2005). Cytoprotective effects of amifostine and cysteamine on cultured normal and tumor cells treated with paclitaxel in terms of mitotic index and 3H-thymidine labeling index. Cancer Chemother. Pharmacol..

[2] Torre LA, Bray F, Siegel RL, Ferlay J, Lortet-Tieulent J, Jemal A (2015). Global cancer statistics. CA Cancer J. Clin..

[3] Siegel RL, Miller KD, Jemal A (2016). Cancer statistics. CA Cancer J. Clin..

[4] Zagouri F, Sergentanis TN, Chrysikos D, Filipits M, Bartsch R (2012). Molecularly targeted therapies in cervical cancer. A systematic review. Gynecol.Oncol..

[5] Yueran LI, Xiao S, Dan L, Xue M (2015). P16INK4A is required for cisplatin resistance in cervical carcinoma SiHa cells. Oncol. Lett..

[6] Peraltazaragoza O, Pérezplasencia C, Salazarleón J, Gómezcerón C, Madridmarina V (2012). Targeted treatments for cervical cancer: a review. Onco Targets Ther..

[7] Jehle T, Bauer J, Blauth E, Hummel A, Darstein M, Freiman TM (2000). Effects of riluzole on electrically evoked neurotransmitter release. Br. J. Pharmacol..

[8] Cheah BC, Vucic S, Krishnan AV, Kiernan MC (2010). Riluzole, neuroprotection and amyotrophic lateral sclerosis. Curr. Med. Chem..

[9] Kitzman PH (2009). Effectiveness of riluzole in suppressing spasticity in the spinal cord injured rat. Neurosci. Lett..

[10] Munro G, Erichsen HK, Mirza NR (2007). Pharmacological comparison of anticonvulsant drugs in animal models of persistent pain and anxiety. Neuropharmacology.

[11] Le MN, Chan JL, Rosenberg SA, Nabatian AS, Merrigan KT, Cohen-Solal KA, Goydos JS (2010). The glutamate release inhibitor riluzole decreases migration, invasion, and proliferation of melanoma cells. J. Invest. Dermatol..

[12] Speyer CL, Nassar MA, Hachem AH, Bukhsh MA, Jafry WS, Khansa RM (2016). Riluzole mediates anti-tumor properties in breast cancer cells independent of metabotropic glutamate receptor-1. Breast Cancer Res. Treat..

[13] Seol HS, Lee SE, Song JS, Lee HY, Park S, Kim I (2016). Glutamate release inhibitor, Riluzole, inhibited proliferation of human hepatocellular carcinoma cells by elevated ROS production. Cancer Lett..

[14] Yelskaya Z, Carrillo V, Dubisz E, Gulzar H, Morgan D, Mahajan SS (2013). Synergistic inhibition of survival, proliferation, and migration of U87 cells with a combination of LY341495 and Iressa. PLoS One.

[15] Zhang C, Yuan XR, Li HY, Zhao ZJ, Liao YW, Wang XY, Su J, Sang SS, Liu Q (2015). Anti-cancer effect of metabotropic glutamate receptor 1 inhibition in human glioma U87 cells: involvement of PI3K/Akt/mTOR pathway. Cell Physiol. Biochem..

[16] Liao S, Ruiz Y, Gulzar H, Yelskaya Z, Taouit LA, Houssou M, Jaikaran T, Schvarts Y, Kozlitina K, Basu-Roy U, Mansukhani A, Mahajan SS (2017). Osteosarcoma cell proliferation and survival requires mGluR5 receptor activity and is blocked by Riluzole. Plos One.

[17] Wu XL, Liu L, Li YJ, Luo J, Gai DW, Lu TL, Mei QB (2018). Synthesis, crystal structure, and antinociceptive effects of some new riluzole derivatives. Med. Chem. Res..

[18] Yuan Z, Zhu T, Zhang X, Jie C, Gang H, Yao H (2015). Role of high-mobility group box 1 in methamphetamine-induced activation and migration of astrocytes. J. Neuroinflammation.

[19] Jamsheena V, Shilpa G, Saranya J, Harry NA, Lankalapalli RS, Priya S (2016). Anticancer activity of synthetic bis (indolyl) methane-ortho-biaryls against human cervical cancer (HeLa) cells. Chem. Biol. Interact..

[20] Farhangi B, Alizadeh AM, Khodayari H, Khodayari S, Dehghan MJ, Khori V, Heidarzadeh A, Khaniki M, Sadeghiezadeh M, Najafi F (2015). Protective effects of dendrosomal curcumin on an animal metastatic breast tumor. Eur. J. Med. Chem..

[21] Wang S, He HB, Xiao SZ, Wang JZ, Bai CH, Wei N, Zou K (2014). Comparison of cardioprotective effects of labeled and unlabeled oleanoic acids with new BOPIM dye on primary neonatal rat cardiomyocytes following hypoxia/reoxygenation injury. Pharmacol. Rep..

[22] Bai C, Yang X, Zou K, He H, Wang J, Qin H, Yu X, Liu C, Zheng J, Cheng F, Chen J (2016). Anti-proliferative effect of RCE-4 from Reineckia carnea on human cervical cancer HeLa cells by inhibiting the PI3K/Akt/mTOR signaling pathway and NF-κB activation. Naunyn. Schmiedebergs Arch. Pharmacol..

[23] Wang Y, Liu P, Qiu L, Sun Y, Zhu M, Gu L, Di W, Duan Y (2013). Toxicity and therapy of cisplatin-loaded EGF modified mPEG-PLGA-PLL nanoparticles for SKOV3 cancer in mice. Biomaterials.

[24] Liang C, Pei S, Ju W, Jia M, Tian D, Tang Y, Mao G (2017). Synthesis and in-vitro and in-vivo antitumour activity study of 11-hydroxyl esterified bergenin/cinnamic acid hybrids. Eur. J. Med. Chem..

[25] Mignani S, Audiau F, Blevec JL (1992). Versatile methods for the synthesis of 2-amino-6-trifluoromethoxy-(nitro) benzothiazoles. Synth. Commun..

[26] Saha P, Ramana T, Purkait N, Ali MA, Paul R (2009). Ligand-free copper-catalyzed synthesis of substituted benzimidazoles, 2-aminobenzimidazoles, 2-aminobenzothiazoles, and benzoxazoles. J. Org. Chem..

[27] Yao GD, Sun Q, Song XY, Huang XX, Song SJ (2018). Flavan enantiomers from Daphne giraldii selectively induce apoptotic cell death in p53-null hepatocarcinoma cells in-vitro. Chem. Biol. Interact..

[28] Machana S, Weerapreeyakul N, Barusrux S, Nonpunya A, Sripanidkulchai B, Thitimetharoch T (2011). Cytotoxic and apoptotic effects of six herbal plants against the human hepatocarcinoma (HepG2) cell line. Chin. Med..

[29] Kamath PR, Sunil D, Joseph MM, Abdul A (2017). A and Srerlekha TT. Indole-coumarin -thiadiazole hybrids: An appraisal of their MCF-7 cell growth inhibition, apoptotic, antimetastatic and computational Bcl-2 binding potential. Eur. J. Med. Chem..

[30] Yin TQ, Yang XO, Jiao FY, Huang LP, Tang XD, Ren BQ (2016). Pseudomonas aeruginosa mannose-sensitive hemagglutinin inhibits proliferation and invasion via the PTEN/AKT pathway in HeLa cells. Oncotarget.

[31] Tong H, Jia L, Xue J, Li H, Xu F, Cheng K, Li D, Li Z, Gao M, Hua H (2017). Scutellarin derivatives as apoptosis inducers: Design, synthesis and biological evaluation. Eur. J. Med. Chem..

[32] Cory S, Adams JM (2005). Killing cancer cells by flipping the Bcl-2/Bax switch. CancerCell..

[33] Adams JM, Cory S (2007). The Bcl-2 apoptotic switch in cancer development and therapy. Oncogene.

[34] Harris MH, Thompson CB (2000). The role of the Bcl-2 family in the regulation of outer mitochondrial membrane permeability. Cell Death Differ..

